# Algorithm for Mobile Platform-Based Real-Time QRS Detection

**DOI:** 10.3390/s23031625

**Published:** 2023-02-02

**Authors:** Luca Neri, Matt T. Oberdier, Antonio Augello, Masahito Suzuki, Ethan Tumarkin, Sujai Jaipalli, Gian Angelo Geminiani, Henry R. Halperin, Claudio Borghi

**Affiliations:** 1 Department of Medicine, Division of Cardiology, Johns Hopkins University, Baltimore, MD 21218, USA; 2 Department of Medical and Surgical Sciences, University of Bologna, 40138 Bologna, Italy; 3 AccYouRate Group S.p.A., 67100 L’Aquila, Italy; 4 Department of Biomedical Engineering, Johns Hopkins University, Baltimore, MD 21218, USA; 5 Department of Radiology, Johns Hopkins University, Baltimore, MD 21205, USA

**Keywords:** wearables, algorithm, electrocardiogram, heart rate, QRS complex, mobile platform

## Abstract

Recent advancements in smart, wearable technologies have allowed the detection of various medical conditions. In particular, continuous collection and real-time analysis of electrocardiogram data have enabled the early identification of pathologic cardiac rhythms. Various algorithms to assess cardiac rhythms have been developed, but these utilize excessive computational power. Therefore, adoption to mobile platforms requires more computationally efficient algorithms that do not sacrifice correctness. This study presents a modified QRS detection algorithm, the AccYouRate Modified Pan–Tompkins (AMPT), which is a simplified version of the well-established Pan–Tompkins algorithm. Using archived ECG data from a variety of publicly available datasets, relative to the Pan–Tompkins, the AMPT algorithm demonstrated improved computational efficiency by 5–20×, while also universally enhancing correctness, both of which favor translation to a mobile platform for continuous, real-time QRS detection.

## 1. Introduction

Continuous collection and real-time analysis of physiologic data are increasingly common due to advances in wearable technologies [1,2]. The electrocardiogram (ECG) is a signal acquisition technology that monitors the electrical activity of the heart and is common for routine cardiac evaluation because it is inexpensive, noninvasive, and provides continuous real-time data. The ECG is particularly valuable for detecting cardiac anomalies such as arrythmias [3,4,5].

Many algorithms have been developed to recognize characteristics of the ECG, and the detection of the QRS complex is fundamental for analysis [6,7,8,9,10,11,12,13,14,15,16,17] because it is the major landmark that allows the waveform to be segmented into heartbeats for determining the heart rate and its variability [18]. The accurate detection of the QRS signal is also fundamental to more detailed ECG processing [19].

One common method for QRS detection is the Pan–Tompkins algorithm, which was developed prior to the advent of wearable technologies [20]. In our experience, the Pan–Tompkins algorithm caused real-time processing delays and was suboptimally correct when evaluating publicly available data that simulated signals that would be acquired from mobile platforms and wearable technologies.

Therefore, to better enable real-time QRS detection via mobile platform-based ECG devices, such as wearable technologies with smartphone interfaces, more computationally efficient algorithms are necessary that do not sacrifice (and may even improve) correctness [21,22,23,24]. We hypothesized that our modified QRS detection algorithm is more computationally efficient and at least as correct as the established method on which it is based.

## 2. Methods

The Pan–Tompkins algorithm was modified (known here as the AccYouRate Modified Pan–Tompkins (AMPT)) to be more computationally efficient and, thus, more amenable to application on a mobile platform. Using archived ECG data from a variety of publicly available ECG datasets, both algorithms were evaluated for computational efficiency and correctness as compared to manually and independently annotated QRS complexes.

### 2.1. Algorithms

The AMPT algorithm differs from the original Pan–Tompkins method in two major ways:

(1) The Pan–Tompkins algorithm performs an analysis on two simultaneous signals: the bandpassed signal and the resulting filtered signal. The peaks from each signal are compared on a time basis for correspondence. However, with the AMPT algorithm, the signal peaks and the noise peaks from the bandpassed signal are not calculated and only the final filtered signal is analyzed.
(2) The Pan–Tompkins algorithm uses two average RR intervals for search back (one for sinus rhythm and one for arrhythmias), which entails defining the signal and noise peaks, thresholds, and a series of RR limits. The AMPT does not differentiate based on regular or irregular rhythms, and therefore requires fewer computational steps because search back is calculated from a single averaged RR interval.

The AMPT algorithm utilizes the same low- and high-pass filters, derivative, squaring function, and moving window integration as the original Pan–Tompkins method. However, only the filtered signal is used for the AMPT. Thus, for the AMPT algorithm, the set of filtered ECG thresholds is redefined as (corresponding to Equations (17)–(20) of the original Pan-Tompkins manuscript [20]):

(1) SPKF=0.125 PEAKF+0.875 SPKF

(2) NPKF=0.125 PEAKF+0.875 NPKF

(3) THRESHOLD F1=NPKF+0.25 (SPKF−NPKF)

(4) THRESHOLD F2=0.25 THRESHOLD F1

where *PEAKF* is the overall peak, *SPKF* is the running estimate of the signal peak, *NPKF* is the running estimate of the noise peak, *THRESHOLD F*1 is the first threshold, and *THRESHOLD F*2 is the second threshold, consistent with nomenclature from the original Pan–Tompkins paper.

Next, when the QRS complex is identified using *THRSHOLD F*2, the signal peak is redefined as (corresponding to Equation (21) of the original Pan-Tompkins manuscript [20]):

(5) SPKF=0.125 PEAKF+0.875 SPKF

Further, the average of the eight most recent sequential *RR* intervals is redefined as (corresponding to Equation (24) of the original Pan-Tompkins manuscript [20]):

(6) RR AVERAGE1=0.125 (RRn−7+RRn−6+…+RRn)

where *RRn* is the most recent *RR* interval. Then, an *RR* limit is redefined as (corresponding to Equation (28) of the original Pan-Tompkins manuscript [20]):

(7) RR MISSED LIMIT=1.66 RR AVERAGE1

The AMPT algorithm also uses the same T-wave identification as the original Pan–Tompkins method.

In summary, from the original Pan–Tompkins manuscript [20], the fiducial mark and Equations (12)–(16), (22), (23), (25)–(27) and (29) were not used, the coefficients of Equations (20) and (21) were modified, and RR Average 1 (rather than RR Average 2) was used in Equation (28) for the AMPT algorithm.

The Pan–Tompkins algorithm was obtained as an intact Python code by Pickus from a public software repository and forum [25]. Via line-by-line inspection, this code was confirmed to exactly implement all steps reported in the original Pan–Tompkins manuscript. The first steps of the algorithm, which include the application of a set of filters, are suitable for the specific, previously used sampling rate of 200 Hz.

The AMPT algorithm was custom written in Python, independent of publicly available Pan–Tompkins codes. The AMPT code is available at https://github.com/Accyourate-Group-S-p-A/acy_ampt (accessed on 25 January 2023).

Both the Pan–Tompkins and AMPT codes were executed in Python 3.7 using IDE PyCharm 2019.2.6 Professional Edition and public Python libraries (NumPy, SciPy, Pandas, WFDB, and time). For comparison purposes, all processing took place on the same desktop computer (HP Z840 Workstation (Hewlett Packard, Palo Alto, CA, USA), Processor: Intel Xeon CPU E5-2620 v3–2.40 GHz, 64 GB RAM (Intel, Santa Clara, CA, USA)) which was only running those background programs (in addition to Python and PyCharm) that loaded upon booting into 64-bit Windows 10 Pro (Microsoft, Redmond, WA, USA).

### 2.2. ECG Datasets

To compare algorithms, multiple datasets were downloaded from the PhysioBank ATM [26] and Harvard Dataverse [27] repositories. Records included annotations of QRS complexes that were manually identified throughout all data and adjudicated independently of this project. The datasets were those curated to specifically feature high and low signal qualities [28], normal sinus rhythms [29], arrhythmias [30], paced rhythms (subset of arrhythmias dataset), and telehealth-acquired signals [27]. The sampling attributes of these datasets are shown in Table 1.

All the data from the High and Low Quality, Arrhythmias, and Paced Rhythm datasets were utilized. However, only the first thirty minutes of each Normal Sinus Rhythm sample were used because when longer periods were considered, hardware resources became a limiting factor and, thus, computational time did not exclusively reflect algorithm efficiency. In addition, from the TeleHealth dataset, 116 samples were excluded because there was a prohibitively small number of reliable ECG waveforms.

Additionally, across datasets and across patients within datasets, there was not a consistent lead configuration. Therefore, in cases where multiple ECG recordings were present, those listed first were utilized.

### 2.3. Analysis

Both algorithms were executed with all samples (except the Normal Sinus Rhythm dataset, from which only the first thirty minutes of each tracing were used), and their outputs were compared to the annotations for each dataset. Accurate detection was indicated if the annotated R peak fell within 150 milliseconds of the algorithm-detected R peak, which is consistent with the ANSI/AAMI guidelines [31]. Other classification possibilities were false positive, false negative, and failed detection. These findings were summarized by calculations for correctness including total error rate, sensitivity, positive predictive value, accuracy, and F1, which were mathematically defined as:

(8) $$Total\;Error\;Rate=\frac{FN+FP}{TB}$$

(9) $$Sensitivity=\frac{TP}{TP+FN}$$

(10) $$Positive\;Predictive\;Value=\frac{TP}{TP+FP}$$

(11) $$Accuracy=\frac{TP}{TP+FP+FN}$$

(12) $$F1=\frac{\left(2*TP\right)}{2*TP+FP+FN}$$

where *FN* is False Negatives (annotated beats that are not detected); *FP* is False Positives (beats detected not corresponding to an annotated beat); *TB* is Total Beats (sum of annotated beats); and *TP* is True Positives (annotated beats that are correctly detected). True negatives are not typically used to calculate the accuracy of QRS detection [7,32]. The processing time of each ECG sample was measured and expressed on the basis of ten seconds of ECG data. A computational efficiency factor was defined as the ratio of times to execute each sample (Pan–Tompkins to AMPT).

For a fair comparison across datasets, the analysis was repeated by resampling all data to 200 Hz. In this way, all ECGs were filtered using the same cut-off frequencies.

## 3. Results

**Figure 1:**
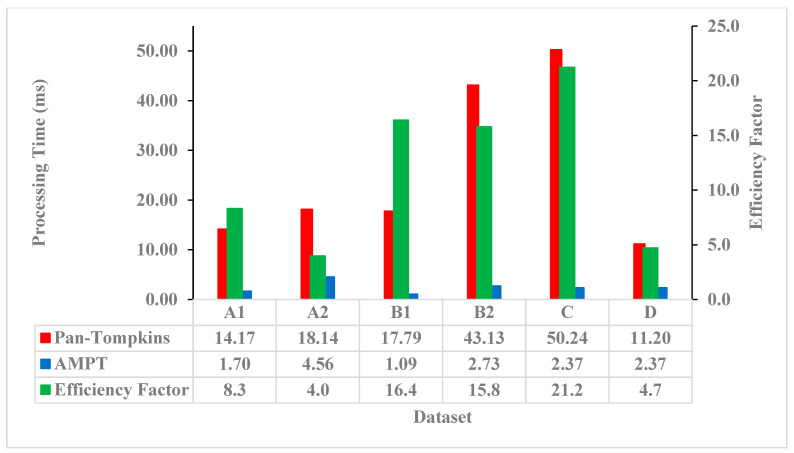
Processing times per ten seconds of ECG data for the Pan–Tompkins (red) and AMPT (blue) algorithms with their efficiency factors (green) by dataset. A1 = High Quality, A2 = Low Quality, B1 = Normal Sinus Rhythm, B2 = Arrhythmias, C = Paced Rhythm, and D = TeleHealth datasets.

As compared to the Pan–Tompkins algorithm, the AMPT algorithm was computationally more efficient across all datasets (Figure 1). For the Pan–Tompkins algorithm, processing times varied from a low of 12.38 milliseconds per ten seconds of ECG data for the TeleHealth dataset to a high of 50.24 milliseconds for the Paced Rhythms dataset, whereas for the AMPT algorithm, the shortest processing time was 1.09 milliseconds per ten seconds of ECG data for the Normal Sinus Rhythm dataset and the longest was 4.56 milliseconds for the Low Quality dataset. As indicated by the efficiency factor and relative to the Pan–Tompkins, the AMPT algorithm improved computational efficiency by a minimum factor of 4.0 for the Low Quality dataset and a maximum of 21.2 for the Paced Rhythms dataset. Intermediate efficiency factors were 8.3, 4.7, 16.4, and 15.8 for the High Quality, TeleHealth, Normal Sinus Rhythms, and Arrhythmias’ datasets, respectively.

**Figure 2:**
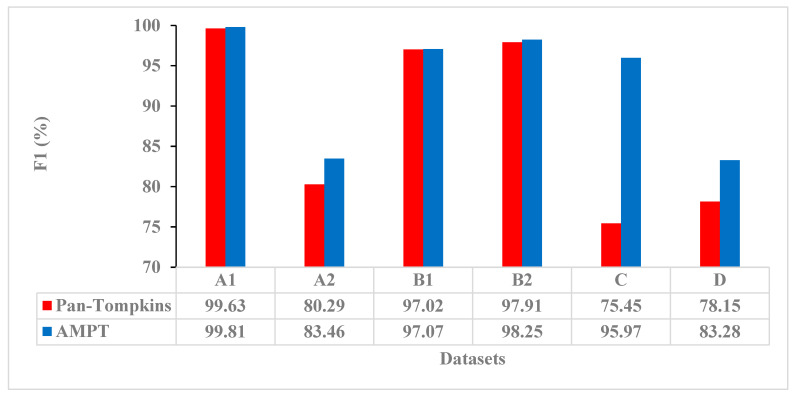
F1 correctness for the Pan–Tompkins (red) and AMPT (blue) algorithms by dataset. A1 = High Quality, A2 = Low Quality, B1 = Normal Sinus Rhythm, B2 = Arrhythmias, C = Paced Rhythm, and D = TeleHealth datasets.

The AMPT algorithm was also more correct than the Pan–Tompkins algorithm according to F1 (Figure 2). For the Pan–Tompkins algorithm, the F1 had a low of 75.45 for the Paced Rhythms dataset and a high of 99.63 for the High Quality dataset, whereas for the AMPT algorithm, the F1 low was 83.28 for the TeleHealth dataset and the high was 99.81 for the High Quality dataset. From the Pan–Tompkins to the AMPT algorithms, the F1 improvement was highest for the Paced Rhythm dataset with a difference of 20.52%. F1 improvements of 3–5% were also demonstrated with the Low Quality and TeleHealth datasets. Across all other measures including error rate, sensitivity, positive predictive value, and accuracy, and for all datasets, the AMPT algorithm was correct more often than the Pan–Tompkins (Table 2).

Within the Arrhythmia dataset, processing times per ten seconds of ECG data and all measures of correctness varied by sample for the Pan–Tompkins and AMPT algorithms (Tables S1 and S2, respectively (Supplementary Materials)). The efficiency gain was less for samples with high amplitude variability or a large number of arrhythmias, and this was determined qualitatively (Figure S1).

After resampling and reprocessing all data at 200 Hz, the relative performance of each algorithm did not change across datasets. The AMPT algorithm was still computationally more efficient and more correct than the original Pan–Tompkins algorithm (Figures S2 and S3). However, the processing times of the resampled data were less than those prior to resampling because there were fewer data points to process.

## 4. Discussion

In evaluating archived and independently annotated ECG data, the AMPT algorithm was both computationally more efficient and more correct than the Pan–Tompkins method. These differences are attributed to the removal of unnecessary, parallel computations, including the double signal analysis for peak detection and one of the two average RR intervals.

Efficiency improvements are dramatic enough to potentially enable the translation of the AMPT algorithm to a mobile platform. However, the AMPT algorithm is relatively less advantageous for processing samples with high amplitude variability or a large number of arrhythmias, as observed by comparing samples with extreme values.

The AMPT algorithm improved F1 correctness by 3–5% for the Low Quality and TeleHealth datasets compared to the Pan–Tompkins, which is significant because these data are most similar to those signals that would be recorded and processed from mobile platforms and wearable technologies. Additionally, with the exception of the sensitivities determined from the Normal Sinus Rhythm dataset, across all dimensions of correctness including total error rate, sensitivity, positive predictive value, accuracy, and F1, the AMPT algorithm outperformed the Pan–Tompkins algorithm for all datasets. Some of the correctness improvements are modest, but they demonstrate that algorithm changes to enhance computational efficiency did not sacrifice accuracy.

AMPT performance was compared to that of Pan–Tompkins; however, it is not possible to directly compare the performance of the AMPT algorithm to other QRS detection algorithms reported in the literature. Inconsistencies in hardware prevent comparison of computational efficiency [7,12,13,14,15,16,17,23], while non-uniform, and unexplained or selective exclusion of data, variations in the temporal width of the detection window, and discrepancies among sampling rates make the direct comparison of accuracies not practical [7,8,9,10,11,17,20,23]. Nonetheless, other studies compare their results to those of Pan–Tompkins [7,8,9,10,11,12,13,14,15,16,17,20,23], and most are faster and more accurate across publicly available datasets, which is consistent with our findings. Therefore, the AMPT would likely be competitive with the computational efficiency and accuracy of other algorithms.

In this analysis, all samples and beats from the High Quality, Low Quality, Arrhythmias, and Paced Rhythms datasets were included because complete sets are most representative of patient or consumer data that would be evaluated in real-time (i.e., continuous analysis of data on a beat-to-beat basis without accumulating a backlog of unanalyzed beats). However, only the first thirty minutes of data from the Normal Sinus Rhythm dataset were studied here because longer periods had excessive computational demands, while thirty minutes per sample was still deemed long enough to determine the relative performance of each algorithm. The exclusion of the TeleHealth samples also does not affect the findings of this study because in most cases, neither algorithm was able to detect the few annotated beats available for analysis in the excluded samples.

The AMPT algorithm was motivated by development of a smart t-shirt that monitors and analyzes a single-lead ECG recording in real-time [33,34]. Initially, the Pan–Tompkins method was utilized with a prototype device; however, there were substantial computational delays and consequent data loss. Therefore, the AMPT algorithm was written to be more computationally efficient, while having improved QRS detection capabilities. In conjunction with the smart t-shirt and on a mobile platform, the AMPT algorithm eliminated the prior delays and data loss and processed the data in real-time without lagging (data not presented).

While demand for computationally efficient ECG analysis algorithms will remain strong due to the need to conserve the energy of battery-powered devices [23], continued advances in mobile device processing speed will diminish the dependency on efficient algorithms to deliver a single ECG analysis outcome. Instead, an efficient QRS detection algorithm will allow for greater complexity of other aspects of parallel ECG analysis such as P- and T-wave analyses [21,22].

This study is limited in that it is a retrospective analysis, and the corresponding patients and pathologies may not represent those of the general population. A more comprehensive future study is necessary to assess the AMPT algorithm’s performance in real-time and with a larger, more diverse patient population. Another limitation is that this study was performed via a desktop computer (rather than on a mobile device) because a consistent platform was necessary to standardize conditions and enable direct comparison of the algorithms’ computational efficiency. Nonetheless, real world application and evaluation will need to feature mobile devices running different operating systems and a variety of other simultaneous applications.

## 5. Conclusions

As compared to the Pan–Tompkins algorithm, the AMPT algorithm demonstrated improved computational efficiency of QRS detection while also enhancing correctness. When applied on a mobile platform, the AMPT algorithm was observed to eliminate processing delays and data loss, which may enable continuous, real-time, single-lead QRS detection on a variety of mobile devices. However, these data were not representative of a clinical trial or field test. Additional studies are necessary to move this technology towards continuous, real-time monitoring of patients and recreational users.

## Figures and Tables

**Table 1:** Number of beats, number of records, record length, total time, and sampling frequency by dataset.

Dataset	Description	Number of Beats	Number of Records	Record Length (min)	Total Time (min)	Sample Frequency (Hz)
A1	High Quality	72,415	100	10	1000	250
A2	Low Quality	78,618	100	10	1000	360
B1	Normal Sinus Rhythm	48,494	18	30	540	128
B2	Arrhythmias	103,724	44	30	1320	360
C	Paced Rhythm	8923	4	30	120	360
D	TeleHealth	6708	134	0.5	125	500

**Table 2:** Correctness of the Pan–Tompkins and AMPT algorithms by dataset. A1 = High Quality, A2 = Low Quality, B1 = Normal Sinus Rhythm, B2 = Arrhythmias, C = Paced Rhythm, and D = TeleHealth datasets.

Dataset	Annotated Peaks	Algorithm	True Positives (Beats)	False Positives (Beats)	False Negatives (Beats)	Failed Detection (Beats)	Error Rate (%)	Sensitivity (%)	Positive Predictive Value (%)	Accuracy (%)
A1	72,415	Pan–Tompkins	72,073	191	348	539	0.74	99.51	99.75	99.26
		AMPT	72,267	135	148	283	0.39	99.80	99.82	99.62
A2	78,618	Pan–Tompkins	64,653	11137	14,065	25,202	32.06	80.75	85.12	74.09
		AMPT	64,993	8671	13,639	22,310	28.38	82.06	86.37	78.54
B1	48,494	Pan–Tompkins	45,231	134	2988	3122	6.44	95.08	99.74	94.85
		AMPT	45,301	8	3083	3091	6.37	94.97	99.98	94.96
B2	103,724	Pan–Tompkins	99,783	349	3720	4069	3.92	96.36	99.66	96.09
		AMPT	100,135	144	3380	3524	3.40	96.80	99.83	96.66
C	8923	Pan–Tompkins	6684	2132	2238	4370	48.97	74.93	75.97	64.26
		AMPT	8468	276	454	730	8.18	96.90	95.07	92.81
D	6708	Pan–Tompkins	3218	972	522	1494	40.10	84.92	77.56	68.91
		AMPT	3394	707	334	1041	27.94	90.00	81.04	76.29

## Data Availability

The data used in this study are openly available in PhysioNet at https://doi.org/10.13026/C2F305 and https://doi.org/10.13026/C2NK5R (accessed on 25 January 2023) for the MIT-BIH arrhythmia and normal sinus rhythm databases, respectively.

## Supplementary Materials

The following supporting information can be downloaded at: https://www.mdpi.com/article/10.3390/s23031625/s1, Figure S1. Representative raw ECG signals for samples 101 (top) and 232 (bottom) of the Arrythmias dataset (B2) visually demonstrating differences in amplitude variability and the number of arrythmias; Figure S2. Processing time per ten seconds of ECG data for the Pan-Tompkins (red) and AMPT (blue) algorithms with their efficiency factors (green) by dataset. A1 = High-Quality, A2 = Low-Quality, B2 = Arrhythmias, C = Paced Rhythm, and D = Telehealth datasets after resampling to 200 Hz; Figure S3. F1 correctness for the Pan-Tompkins (red) and AMPT (blue) algorithms by dataset. A1 = High-Quality, A2 = Low-Quality, B2 = Arrhythmias, C = Paced Rhythm, and D = Telehealth datasets after resampling to 200 Hz; Table S1. Processing time per ten seconds of ECG data and correctness of the Pan-Tompkins algorithm for the Arrhythmias dataset (B2) by sample; Table S2. Processing time per ten seconds of ECG data and correctness of the AMPT algorithm for the Arrhythmias dataset (B2) by sample.
