# Supplementary material for: Algorithm for Mobile Platform-Based Real-Time QRS Detection
Source: Sensors (Basel). 2023 Feb 2;23(3):1625. doi: 10.3390/s23031625 (PMC9920820; doi:10.3390/s23031625)
Supplement: Supplementary file 1 [file sensors-23-01625-s001.zip › sensors-2058026-supplementary.pdf]

Supplement

Tables

Table S1. Processing time per ten seconds of ECG data and correctness of the Pan-Tompkins algorithm for the Arrhythmias dataset (B2) by sample.

Sample	Processing time (ms)	Total (Annotated Beats)	Peaks Detected	True Positives	False Positives	False Negatives	Failed Detection (Beats)	Total Error Rate (%)	Sensitivity (%)	Positive Predictive Value (%)	Accuracy (%)	F1 (%)
100	42.71	2274	2270	2270	0	4	4	0.18	99.82	100.00	99.82	99.91
101	38.71	1874	1866	1865	1	9	10	0.53	99.52	99.95	99.52	99.73
103	39.15	2091	2080	2080	0	11	11	0.53	99.47	100.00	99.47	99.74
105	40.11	2691	2604	2556	48	115	163	6.06	95.69	98.16	95.69	96.91
106	42.18	2098	2016	2016	0	82	82	3.91	96.09	100.00	96.09	98.01
108	40.71	1824	1778	1755	23	59	82	4.50	96.75	98.71	96.75	97.72
109	53.47	2535	2523	2523	0	12	12	0.47	99.53	100.00	99.53	99.76
111	46.44	2133	2121	2118	3	15	18	0.84	99.30	99.86	99.30	99.58
112	45.69	2550	2536	2535	1	15	16	0.63	99.41	99.96	99.41	99.69
113	36.80	1796	1792	1792	0	4	4	0.22	99.78	100.00	99.78	99.89
114	37.58	1890	1426	1384	42	506	548	28.99	73.23	97.05	73.23	83.47
115	39.93	1962	1950	1950	0	10	10	0.51	99.49	100.00	99.49	99.74
116	45.40	2421	2387	2386	1	35	36	1.49	98.55	99.96	98.55	99.25
117	35.67	1539	1532	1532	0	7	7	0.45	99.55	100.00	99.55	99.77
118	44.88	2301	2276	2275	1	25	26	1.13	98.91	99.96	98.91	99.43
119	39.75	2094	1984	1984	0	110	110	5.25	94.75	100.00	94.75	97.30
121	36.72	1876	1859	1859	0	14	14	0.75	99.25	100.00	99.25	99.62
122	38.10	2479	2472	2472	0	6	6	0.24	99.76	100.00	99.76	99.88
123	34.03	1519	1515	1515	0	4	4	0.26	99.74	100.00	99.74	99.87
124	41.84	1634	1606	1606	0	28	28	1.71	98.29	100.00	98.29	99.14
200	44.88	2792	2598	2593	5	197	202	7.23	92.94	99.81	92.94	96.25
201	39.06	2039	1894	1894	0	145	145	7.11	92.89	100.00	92.89	96.31
202	41.12	2146	2117	2117	0	28	28	1.30	98.69	100.00	98.69	99.34
203	50.60	3108	2914	2897	17	175	192	6.18	94.30	99.42	94.30	96.79
205	44.27	2672	2650	2648	2	17	19	0.71	99.36	99.92	99.36	99.64
207	54.98	2385	2170	2152	18	227	245	10.27	90.46	99.17	90.46	94.61
208	47.65	3040	2868	2717	151	317	468	15.39	89.55	94.74	89.55	92.07
209	50.26	3052	3003	3002	1	43	44	1.44	98.59	99.97	98.59	99.27
210	46.52	2685	2580	2574	6	108	114	4.25	95.97	99.77	95.97	97.83
212	45.05	2763	2747	2743	4	18	22	0.80	99.35	99.85	99.35	99.60
213	45.22	3294	3242	3242	0	52	52	1.58	98.42	100.00	98.42	99.20
214	42.10	2297	2255	2253	2	43	45	1.96	98.13	99.91	98.13	99.01
215	52.60	3400	3351	3351	0	36	36	1.06	98.94	100.00	98.94	99.47
219	47.48	2312	2151	2151	0	89	89	3.85	96.03	100.00	96.03	97.97
220	36.98	2069	2044	2044	0	25	25	1.21	98.79	100.00	98.79	99.39
221	42.44	2462	2419	2419	0	40	40	1.62	98.37	100.00	98.37	99.18
222	45.57	2634	2475	2466	9	155	164	6.23	94.09	99.64	94.09	96.78
223	43.05	2643	2590	2590	0	53	53	2.01	97.99	100.00	97.99	98.99
228	44.79	2141	2060	2047	13	91	104	4.86	95.74	99.37	95.74	97.52
230	40.27	2466	2252	2252	0	213	213	8.64	91.36	100.00	91.36	95.48
231	36.80	2011	1568	1568	0	443	443	22.03	77.97	100.00	77.97	87.62
232	45.92	1816	1779	1778	1	33	34	1.87	98.18	99.94	98.18	99.05
233	51.65	3152	3068	3068	0	82	82	2.60	97.40	100.00	97.40	98.68
234	38.54	2764	2744	2744	0	19	19	0.69	99.31	100.00	99.31	99.65
**Totals**	**1897.67**	**103724**	**100132**	**99783**	**349**	**3720**	**4069**	**3.92**	**96.36**	**99.66**	**96.36**	**97.91**

Table S2. Processing time per ten seconds of ECG data and correctness of the AMPT algorithm for the Arrhythmias dataset (B2) by sample.

Sample	Processing time (ms)	Total (Annotated Beats)	Peaks Detected	True Positives	False Positives	False Negatives	Failed Detection (Beats)	Total Error Rate (%)	Sensitivity (%)	Positive Predictive Value (%)	Accuracy (%)	F1 (%)
100	2.69	2274	2271	2271	0	3	3	0.13	99.87	100.00	99.87	99.93
101	1.91	1874	1865	1863	2	10	12	0.64	99.47	99.89	99.36	99.68
103	2.00	2091	2084	2084	0	7	7	0.33	99.67	100.00	99.67	99.83
105	1.91	2691	2577	2555	22	114	136	5.05	95.73	99.15	94.95	97.41
106	2.26	2098	2026	2026	0	72	72	3.43	96.57	100.00	96.57	98.25
108	2.17	1824	1762	1682	80	134	214	11.73	92.62	95.46	88.71	94.02
109	2.00	2535	2530	2530	0	5	5	0.20	99.80	100.00	99.80	99.90
111	2.26	2133	2123	2123	0	9	9	0.42	99.58	100.00	99.58	99.79
112	2.26	2550	2539	2539	0	10	10	0.39	99.61	100.00	99.61	99.80
113	1.82	1796	1794	1794	0	2	2	0.11	99.89	100.00	99.89	99.94
114	3.56	1890	1855	1849	6	41	47	2.49	97.83	99.68	97.52	98.74
115	1.82	1962	1952	1952	0	8	8	0.41	99.59	100.00	99.59	99.80
116	2.60	2421	2391	2389	2	32	34	1.40	98.68	99.92	98.60	99.29
117	2.00	1539	1534	1534	0	5	5	0.32	99.68	100.00	99.68	99.84
118	2.60	2301	2277	2277	0	21	21	0.91	99.09	100.00	99.09	99.54
119	2.08	2094	1987	1987	0	107	107	5.11	94.89	100.00	94.89	97.38
121	1.91	1876	1862	1862	0	11	11	0.59	99.41	100.00	99.41	99.71
122	2.00	2479	2475	2475	0	3	3	0.12	99.88	100.00	99.88	99.94
123	2.17	1519	1514	1514	0	5	5	0.33	99.67	100.00	99.67	99.84
124	1.91	1634	1619	1619	0	15	15	0.92	99.08	100.00	99.08	99.54
200	2.17	2792	2597	2596	1	192	193	6.91	93.11	99.96	93.08	96.42
201	3.12	2039	1908	1908	0	131	131	6.42	93.58	100.00	93.58	96.68
202	2.17	2146	2131	2131	0	14	14	0.65	99.35	100.00	99.35	99.67
203	3.65	3108	2708	2703	5	376	381	12.26	87.79	99.82	87.65	93.42
205	2.00	2672	2636	2636	0	32	32	1.20	98.80	100.00	98.80	99.40
207	3.47	2385	2101	2093	8	278	286	11.99	88.27	99.62	87.98	93.60
208	3.73	3040	2908	2904	4	130	134	4.41	95.72	99.86	95.59	97.74
209	2.00	3052	2986	2986	0	56	56	1.83	98.16	100.00	98.16	99.07
210	2.08	2685	2580	2579	1	103	104	3.87	96.16	99.96	96.12	98.02
212	2.08	2763	2747	2747	0	14	14	0.51	99.49	100.00	99.49	99.75
213	2.43	3294	3246	3246	0	48	48	1.46	98.54	100.00	98.54	99.27
214	1.91	2297	2257	2257	0	39	39	1.70	98.30	100.00	98.30	99.14
215	2.26	3400	3342	3342	0	49	49	1.44	98.55	100.00	98.55	99.27
219	2.43	2312	2147	2147	0	115	115	4.97	94.92	100.00	94.92	97.39
220	2.00	2069	2045	2045	0	24	24	1.16	98.84	100.00	98.84	99.42
221	2.34	2462	2409	2409	0	49	49	1.99	98.01	100.00	98.01	98.99
222	4.60	2634	2422	2414	8	205	213	8.09	92.17	99.67	91.89	95.77
223	2.17	2643	2602	2602	0	41	41	1.55	98.45	100.00	98.45	99.22
228	2.78	2141	2046	2045	1	89	90	4.20	95.83	99.95	95.78	97.85
230	2.43	2466	2255	2255	0	210	210	8.52	91.48	100.00	91.48	95.55
231	2.26	2011	1571	1571	0	440	440	21.88	78.12	100.00	78.12	87.72
232	18.23	1816	1781	1777	4	33	37	2.04	98.18	99.78	97.96	98.97
233	2.08	3152	3065	3065	0	87	87	2.76	97.24	100.00	97.24	98.60
234	1.82	2764	2752	2752	0	11	11	0.40	99.60	100.00	99.60	99.80
**Totals**	**120.13**	**103724**	**100279**	**100135**	**144**	**3380**	**3524**	**3.40**	**96.80**	**99.83**	**96.66**	**98.25**

Figure

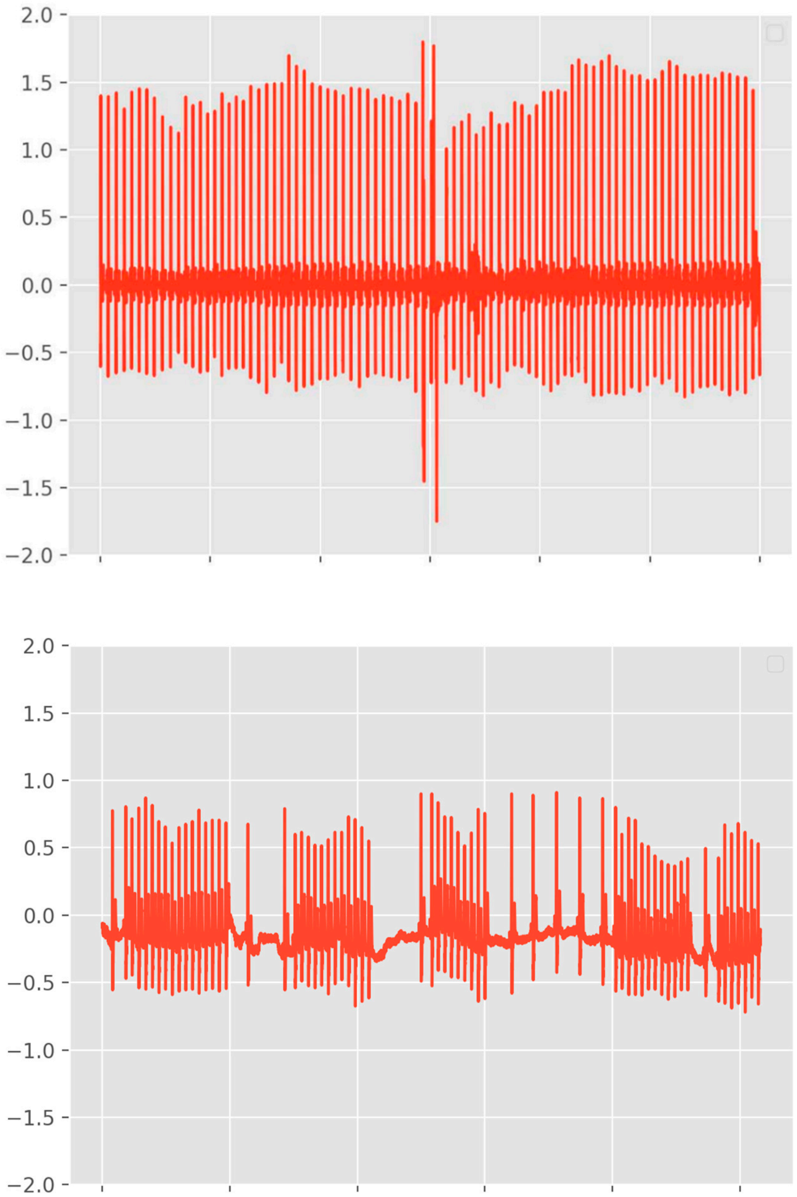

Figure S1. Representative raw ECG signals for samples 101 (top) and 232 (bottom) of the Arrythmias dataset (B2) visually demonstrating differences in amplitude variability and the number of arrythmias.

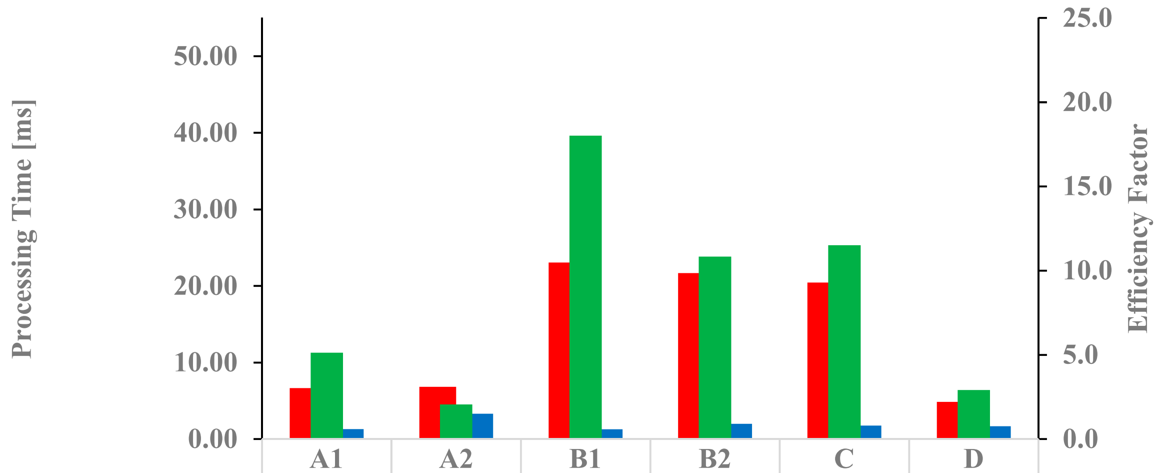

	A1	A2	B1	B2	C	D
Pan-Tompkins	6.66	6.83	23.06	21.67	20.44	4.87
AMPT	1.30	3.33	1.28	2.00	1.78	1.67
Efficiency Factor	5.12	2.05	18.02	10.83	11.50	2.92

Dataset

Figure S2. Processing time per ten seconds of ECG data for the Pan-Tompkins (red) and AMPT (blue) algorithms with their efficiency factors (green) by dataset. A1 = High-Quality, A2 = Low-Quality, B2 = Arrhythmias, C = Paced Rhythm, and D = Telehealth datasets after resampling to 200 Hz.

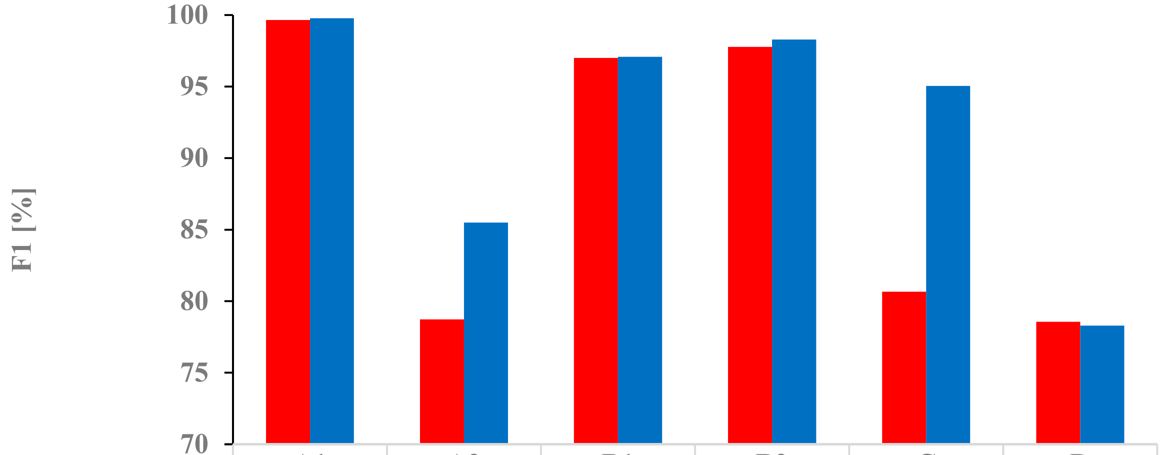

	A1	A2	B1	B2	C	D
Pan-Tompkins	99.65	78.73	97.00	97.77	80.67	78.56
AMPT	99.78	85.49	97.08	98.28	95.04	78.30

Dataset

Figure S3. F1 correctness for the Pan-Tompkins (red) and AMPT (blue) algorithms by dataset. A1 = High-Quality, A2 = Low-Quality, B2 = Arrhythmias, C = Paced Rhythm, and D = Telehealth datasets after resampling to 200 Hz.
